# "Is Cybermedicine Killing You?" - Peer Review and Evidence-Based Medicine

**DOI:** 10.2196/jmir.7.4.e38

**Published:** 2005-07-28

**Authors:** Joshua Fogel

A recent JMIR article [1] and corresponding editorial [2] discuss an error in a review by a Cochrane Collaboration Group [3]. The articles accurately demonstrate that an error occurred, with the traditional approach to peer review failing.

A solution to this situation is necessary as similar errors could occur in the future. The Cochrane Collaboration attempts to achieve a higher standard than systematic review articles and meta-analysis articles published in other journals. The Cochrane Library claims it is "the *best* [italics added] single source of reliable evidence about the effects of health care" [4]. Striving for "the best" should include following best practices for peer review.

Rada [1] advocates extending the Cochrane Collaboration's current practice of open commentary to the prepublication phase. Articles could only be published once there has been extensive commenting by any interested individuals and a consensus has been achieved. Although a good suggestion, there are a few concerns. First, how much time would be necessary before a review period would be deemed appropriate and the article is published? Second, it often can be impossible to reach a consensus among all the reviewers, especially if there were a large number of individuals commenting on a particular topic.

Eysenbach and Kummervold [2] recommend making it a requirement to invite all primary authors quoted in the systematic review to comment on the review before publication. This suggestion has a lot of merit as this would guarantee that some of the peer reviewers are not only knowledgeable scientists, but also actual experts in the specific topic reviewed.

I suggest taking this a step further. The current Cochrane Collaboration policy is to have 4 peer reviewers for each manuscript [2]. My suggestion is that 2 peer reviewers should be specifically among those whose primary studies have been quoted. One should be from a positive outcome study and the other from a negative outcome study. This would give fair representation to each side on the topic being reviewed. The 2 other peer reviewers could be knowledgeable scientists who are not quoted in the review. These 2 other peer reviewers would be no different than the current standard for a typical journal article that usually has 2 peer reviewers.

Furthermore, each Cochrane review should state the level of the peer review on the title page. For example, a level "A" review would be a review with 4 peer reviews. There would be 1 reviewer from a positive outcome study quoted in the primary review, 1 reviewer from a negative outcome study quoted in the primary review, and 2 additional reviewers who have not been quoted in the review. A level "B1" review would have 4 peer reviews, similar to level "A," but would have only 1 peer reviewer from a primary positive outcome study review the manuscript. A level "B2" review would have 4 peer reviews, similar to level "A," but would have only 1 peer reviewer from a primary negative outcome study review the manuscript. Finally, a level "C" review would have 4 peer reviews but would not have any peer reviewers whose studies were quoted in the primary review.

This approach for peer review may prevent future errors from occurring and maintain Cochrane Collaboration articles as the standard for systematic reviews.
